# 2393. Factors Associated with Time to COVID-19 Vaccination among People with HIV

**DOI:** 10.1093/ofid/ofad500.2013

**Published:** 2023-11-27

**Authors:** Aasith Villavicencio Paz, Alex P Sanchez-Covarrubias, Fatemeh Ghadimi, Hervette Nkwihoreze, Raymond Zelada, Helen Koenig, Florence Momplaisir

**Affiliations:** Hospital of the University of Pennsylvania, Philadelphia, Pennsylvania; University of Miami, Miller School of Medicine, Miami, Florida; University of Pennsylvania, Bronx, New York; University of Pennsylvania, Bronx, New York; University of Rochester, Rochester, New York; Hospital of the University of Pennsylvania, Philadelphia, Pennsylvania; University of Pennsylvania, Bronx, New York

## Abstract

**Background:**

The COVID-19 pandemic has significantly impacted at-risk populations, including people with HIV/AIDS (PWHA). Several reports indicate a higher risk of SARS-CoV-2 infection and mortality in PWHA compared to the general population. Despite an effective vaccine, PWHA with low CD4 count develop a weak immune response and are at risk of severe breakthrough infections. Vaccination gaps have been identified amongst PWHA, but factors associated with time to mRNA COVID-19 vaccine initiation and first booster have not been studied.

**Methods:**

PWHA age ≥ 18 years, in HIV care at an urban clinic, and who completed the primary series of COVID-19 vaccines were invited to complete an online survey. Socio-demographic and clinical characteristics and COVID-19 vaccination motivators were assessed and complemented by a review of medical records. Statements on motivators to vaccination were rated on a 5-point Likert scale treated as ordinal data (minimum score 19, maximum 95). Factors associated with time to first COVID-19 vaccine and first booster were analyzed by the Kaplan-Meier method.

**Results:**

A total of 129 patients enrolled; 83% (108/129) received the COVID-19 booster. Mean age was 50.3 (±10.9) years; 26.4% were female; 54.3% were Black, and 34.1% were White; 91.5% were non-Hispanic. The mean nadir CD4 count throughout their care was 309.4 (±245.6) cells/mm3. The mean time to first COVID-19 vaccine dose was 2.8 (±1.8) months from December 2020 (Pfizer-BioNTech vaccine approval), and time to first booster was 9.9 (±3.8) months from the date of the first vaccine. Factors associated with quicker uptake of the first dose included being ≥ 51 years of age (p=0.001), White race (p=0.027), male sex (p=0.023), and gay/lesbian identity (p=0.014) (Fig 1). Factors associated with quicker uptake of the first booster included being ≥ 51 years of age (p < 0.000) and a COVID-19 motivators’ survey score > 69 (p=0.017) (Fig 2).

Figure 1.

**Figure:**
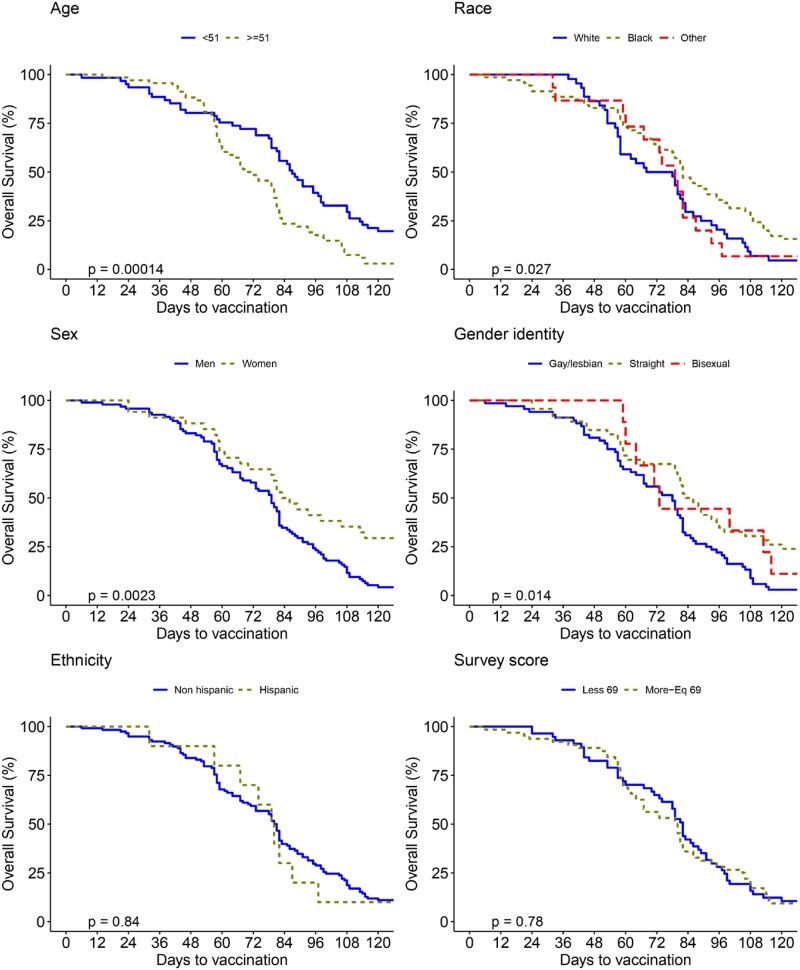

Kaplan-Meier Curves for Time to COVID-19 First Dose Vaccination Among People Living with HIV/AIDS Receiving HIV Care through Penn Medicine.

Figure 2.

**Figure:**
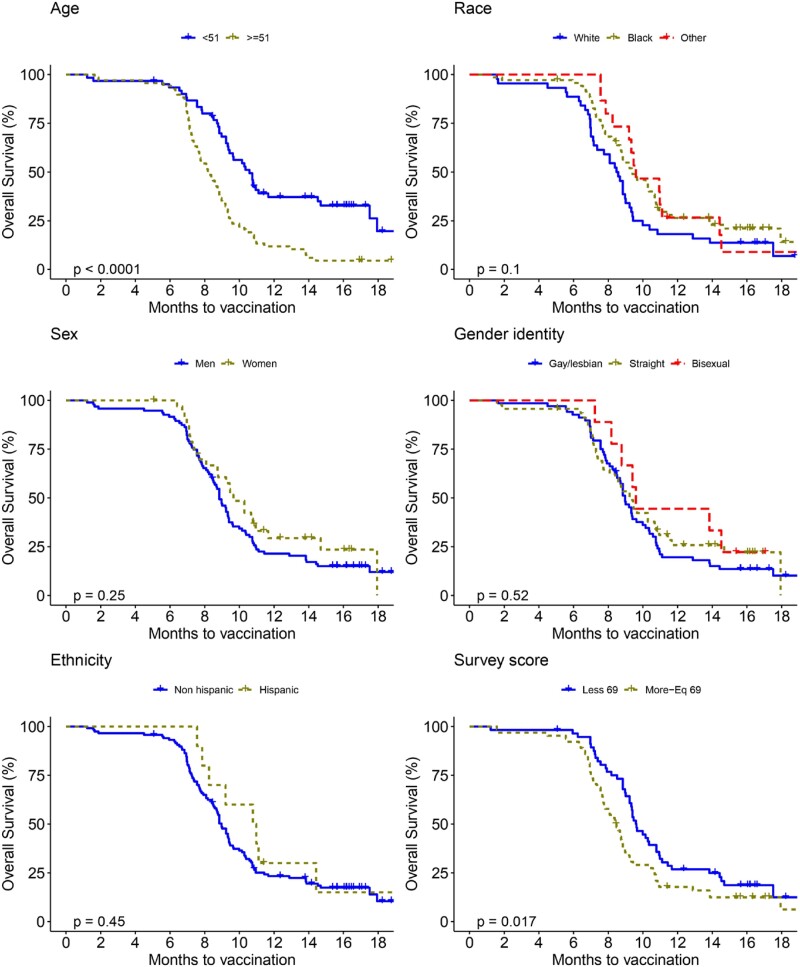

Kaplan-Meier Curves for Time to COVID-19 First Booster Vaccination Among People Living with HIV/AIDS Receiving HIV Care through Penn Medicine.

**Conclusion:**

We found high rates of COVID-19 booster vaccination among PWHA. White and male patients got vaccinated quicker in the early phase of the pandemic; however, race and age-based inequities resolved in the later phase of the pandemic. Motivation to vaccination emerged as a facilitator for uptake of booster vaccines.

**Disclosures:**

**All Authors**: No reported disclosures

